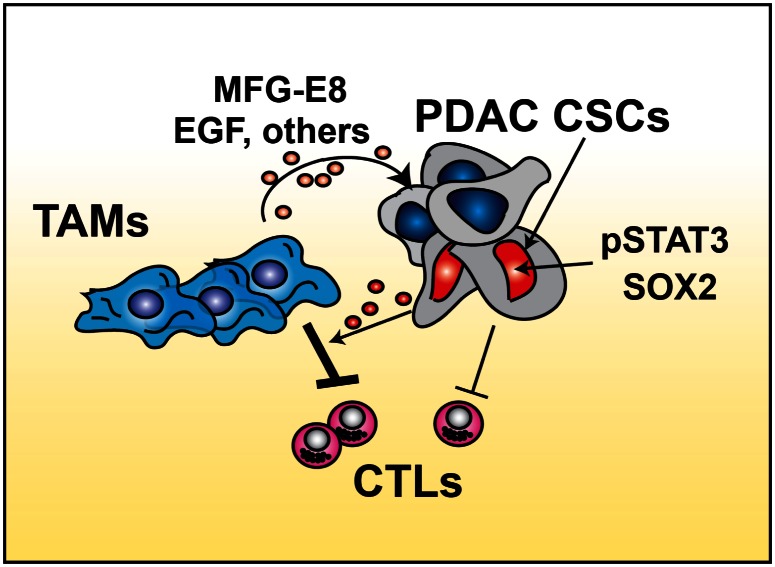# Tumor-infiltrating macrophages, cancer stem cells and therapeutic responses

**DOI:** 10.18632/oncotarget.792

**Published:** 2012-12-31

**Authors:** Roheena Z. Panni, David C. Linehan, David G. DeNardo

**Affiliations:** Department of Surgery, Washington University School of Medicine, St Louis, MO, USA; Departments of Surgery, Siteman Cancer Center, Washington University School of Medicine, St Louis, MO, USA; Departments of Medicine, Pathology and Immunology and Siteman Cancer Center, Washington University School of Medicine, St Louis, MO, USA

While the genetic and epigenetic changes that regulate cell proliferation, survival and/or differentiation are known ‘initiators’ of tumor development, these events do not occur in isolation but rather, in the context of a diverse organ specific tumor stroma. Of the stromal components, tumor-infiltrating immune cells are a hallmark of most solid tumors, and the presence of varied immune populations can significantly affect clinical outcomes for cancer patients. Historically, tumor-infiltrating immune cells have been viewed as restraining tumor progression, but in recent years, this view has been expanded to appreciate that chronic immune responses also play critical roles in promoting tumor progression, metastasis, and resistance to cytotoxic therapies[1]. Therefore, understanding the molecular mechanisms by which malignant cells derail antitumor immune responses to favor disease progression is critical to identify potential therapeutic targets. In addition to immune regulation of cancer progression and chemoresistance, tumor cells that acquire stem-like or tumor-initiating properties (often called “Cancer Stem Cells”) exhibit enhanced resistance to cytotoxic therapy and increased propensity for metastatic dissemination[2]. Several lines of evidence suggest that the tumor-initiating capacity of malignant cells is rooted in inflammatory signals [3]. However, the mechanisms by which different populations of leukocytes might regulate cancer stem cells (CSCs) are unknown. Recently, three studies have demonstrated that tumor-infiltrating macrophages (TAMs) enhance tumor-initiating properties in malignant cells and that these regulatory pathways can be therapeutically exploited.

Work by Tahara *et al*. identified Milk Fat Globulin Epidermal growth factor-8 (MFGE-8) as a macrophage derived factor, which potently increase the tumor initiating properties of murine Colon and Lung Carcinoma cell lines[4]. This activity was attributed to both activation of Signal transducer and activator of transcription 3 (STAT3) signaling and enhancement of the Hedgehog signaling. These pathways are major contributors in triggering tumorigenicity and resistance to anti-cancer therapy. They also found that IL-6 co-ordinates with MFGE8 and plays a critical role in increasing tumorigenic activities in subsets of CSCs of primary human tumors. These data suggest that TAMs play a key role in CSC maintenance and/or expansion. Similar work by Yunping Luo *et al*. found that the crosstalk between TAMs and tumor cells can regulate the induction of pluripotency gene SOX-2 through EGFR mediated activation of STAT3 signaling[5]. Their data also suggests that inhibition of EGF or STAT3 prevents the up regulation of SOX-2 and thus improves chemo-sensitivity in human patients.

An alternative approach to targeting these signaling pathways is to block the recruitment or bioactivity of the tumor-infiltrating macrophages directly. Work from several research groups have shown the potential efficacy in targeting macrophages and monocytes through either Colony Stimulating Factor 1 Receptor (CSF1R) or C-C chemokine receptor 2 (CCR2) to block tumor progression, metastasis and/or improve response to chemotherapy [6, 7]. This research has spurred several new clinical trials of agents targeting macrophages through inhibition of these receptors or their ligands. However, the impact of such therapies on CSCs is not well understood. Recent work by Mitchem *et al*. showed that blockade of CCR2 or CSF1R signaling in established tumors not only results in a significant decrease in tumor infiltration by macrophages, but also reduces the frequency of CD44+ALDH1+ pancreatic CSCs[8]. According to their results, targeting macrophages through CSF1R blockade also improved response to chemotherapy. Additionally, while treatment with single-agent Gemcitabine, CCR2i, or CSF1Ri did not alter T-cell infiltration but the combination therapy significantly improved the T cell immune response, suggesting that both tumor cell destruction and macrophage depletion are necessary to sustain cytotoxic T lymphocyte (CTL) infiltration in pancreatic ductal adenocarcinoma (PDAC).

Consistent with the studies in mammary and colon cancer cells, Mitchem et al. found that TAMs can directly induce CSC properties and chemo-resistance in PDAC cells through the activation of STAT3 *in-vitro* and that targeting CSF1R or CCR2 decreases STAT3 signaling in-vivo. Interestingly, previous studies have demonstrated that inhibition of STAT3 signaling can increase anti-tumor immune responses by T cells, suggesting that there might be a connection between STAT3, CSCs and CTL responses. Supporting this possible connection, Mitchem *et al* observed that CD44^+^ALDH^Bright^ PDAC CSCs induce immunosuppressive behavior in TAMs in a STAT3 dependent manner. Taken together, with the observation that macrophage depletion in combination with chemo can induce CTL responses[6, 8], these data together suggest that crosstalk between TAMs and CSCs may enhance immune suppression and thus blunt anti-tumor T cell activity during chemotherapy (Figure 1). Thus the results from these three groups suggest that reprograming of the immune microenvironment might be an effective way to target CSCs and improve outcomes for high-risk patients.

**Figure:**